# A high-throughput screening platform to identify *MYCN* expression inhibitors for liver cancer therapy

**DOI:** 10.3389/fonc.2025.1486671

**Published:** 2025-02-14

**Authors:** Yali Xu, Hricha Mishra, Yutaka Furutani, Kaori Yanaka, Hajime Nishimura, Erina Furuhata, Masataka Takahashi, Luc Gailhouste, Yusuke Suenaga, Yoshitaka Hippo, Wenkui Yu, Tomokazu Matsuura, Harukazu Suzuki, Xian-Yang Qin

**Affiliations:** ^1^ Laboratory for Cellular Function Conversion Technology, RIKEN Center for Integrative Medical Sciences, Kanagawa, Yokohama, Japan; ^2^ Department of Intensive Care Unit, The Affiliated Drum Tower Hospital, Medical School of Nanjing University, Nanjing, Jiangsu, China; ^3^ Department of Laboratory Medicine, The Jikei University School of Medicine, Tokyo, Japan; ^4^ Laboratory for Brain Development and Disorders, RIKEN Center for Brain Science, Saitama, Japan; ^5^ Laboratory of Evolutionary Oncology, Chiba Cancer Center Research Institute, Chiba, Japan

**Keywords:** MYCN, liver cancer, high-throughput screening, DDI core library, gRNA library, Acot2

## Abstract

MYCN, an oncogene implicated in hepatocellular carcinoma (HCC), is predominantly expressed in cancer stem-like HCC cells. It drives tumorigenicity, metastasis, and therapeutic resistance. In this study, we hypothesized that the pharmacological inhibition of MYCN could represent a novel therapeutic strategy for HCC. To identify inhibitors of MYCN expression, we developed an unbiased, high-throughput screening platform. With this platform, we identified MI202 as a potent inhibitor of *MYCN* expression. MI202 significantly reduced *MYCN* promoter activity and mRNA levels in HCC cells, inhibiting cell proliferation, spheroid formation, and colony growth and promoting apoptosis. Notably, MI202 selectively inhibited the proliferation of HCC cells but not of normal hepatic cells, highlighting its potential for HCC-specific therapy. Genome-wide CRISPR knockout screening has identified acyl-CoA thioesterase 2 (*ACOT2*), a key regulator of lipid metabolism, as a molecular target of MI202. *ACOT2* downregulation by MI202 was associated with reduced MYCN expression, suggesting that ACOT2 may mediate MYCN-driven tumorigenesis through lipid desaturation. Overall, this study presents a robust high-throughput screening platform to identify MYCN inhibitors and highlights the potential of pharmacological downregulation of MYCN as a therapeutic strategy for targeting HCC.

## Introduction

The incidence of liver cancer has been increasing over the past two decades, and it is estimated that more than one million individuals will be affected annually by 2025 (1, 2). Hepatocellular carcinoma (HCC), the most common primary liver cancer, is the fourth leading cause of cancer-related deaths worldwide (3). Despite significant advances in personalized therapies and immunotherapy, this malignancy is still associated with high recurrence rates due to the self-renewal and high tumorigenic potential of liver cancer stem cells (CSCs) (1, 4). Notably, the prevalence of HCC among patients with non-viral etiologies, such as metabolic syndrome and diabetes, continues to rise in Japan, highlighting the critical role of lipid metabolism in liver cancer (5, 6). Particularly, there is growing concern regarding the role of lipid desaturation in maintaining CSC stemness by regulating Wnt/β-catenin signaling and endoplasmic reticulum (ER) stress pathways (7–9).

*MYCN* is a member of the Myc proto-oncogene family (10). It encodes the transcription factor MYCN, which regulates genes involved in biological processes required for tumorigenesis, such as cell growth, proliferation, differentiation, and genome stability (10). Amplification and concomitant overexpression of the *MYCN* oncogene are commonly observed in nervous system malignancies such as neuroblastoma and medulloblastoma and are frequently associated with poor prognosis (11). Recently, both our group and the others have elucidated the oncogenic roles of MYCN in the tumorigenesis and progression of non-neuronal tumors, including HCC (12–15). *MYCN* is upregulated in HCC cells and tumor tissues from patients with HCC and is correlated with the recurrence of *de novo* HCC (16). Unlike c-MYC, MYCN is expressed in a specialized EpCAM-positive CSC-like subpopulation of heterogeneous HCC cells, which is selectively eliminated by a candidate chemopreventive agent against HCC recurrence, acyclic retinoid (ACR) (16, 17). Notably, increased MYCN protein levels were observed in the serum of HCC patients compared to healthy controls and were associated with liver functional reserve, fibrosis, and long-term prognosis (14). A retrospective analysis of a phase 3 clinical trial of ACR indicated that serum MYCN levels could help identify HCC patients who may benefit from ACR treatment. Patients with reduced serum MYCN protein levels show a better prognosis after ACR treatment compared to patients with increased serum MYCN protein levels (14). In addition, the loss of MYCN function suppresses the growth and invasion of HCC cells (16, 18). Additionally, there is growing concern about the role of MYCN in metabolic reprogramming in cancer cells (19). MYCN promotes fatty acid synthesis by cooperating with mondo A–sterol regulatory element-binding protein 1 (SREBP1) or directly activating transcription of key lipogenic enzymes, including acetyl-CoA carboxylase (ACC), fatty acid synthase (FASN), and stearoyl-CoA desaturase (SCD1) (15, 20–22). Collectively, these studies suggest that inhibition of MYCN and MYCN-driven lipid metabolic dependency could be a promising strategy for the prevention and treatment of HCC.

MYCN regulation encompasses various molecular interactions and modifications, including histone mutations (23, 24) and coactivator interactions (25) in the context of brain tumors. Drugs and small molecular inhibitors have been developed to target MYCN and its associated pathways (26–29). Direct strategies include inhibiting MYCN transcription using bromodomain and extra-terminal (BET) inhibitors or disrupting its DNA-binding function (26, 30, 31). Indirect approaches involve modulating MYCN activity by targeting synthetic lethal interactions with proteins such as AURKA and CDK1 or destabilizing MYCN by disrupting its oncogenic stabilization (32). Furthermore, targeting downstream regulatory genes of MYCN, such as ornithine decarboxylase 1 (*ODC1*) or the transcriptional machinery it regulates presents additional therapeutic opportunities (33). Notably, inhibitors such as the BET protein inhibitor GSK525762, the AURKA inhibitor MLN8237, and the ODC inhibitor DFMO are currently undergoing clinical evaluation for MYCN-driven cancers. Despite these advances, effectively targeting MYCN remains a significant challenge due to its lack of a defined active site, intrinsically disordered regions, and the adaptability of transcriptional programs in cancer cells. As the transcription of *MYCN* represents a critical initiating event in its oncogenic activation, suppressing this process may substantially enhance the efficacy of MYCN-targeted therapies (34). In this study, we conducted a high-throughput screening using HCC cells engineered with an *MYCN* promoter-reporter to identify novel small molecules that downregulate *MYCN* promoter activity and gene expression. We identified MI202 (1-benzothiazol-2-yl-1h-pyrrole-2-carbaldehyde) as a hit compound that suppresses *MYCN* expression in HCC cells. Notably, MI202 selectively inhibited HCC cell proliferation while having a limited effect on normal hepatic cells. Using CRISPR/Cas9-based whole-genome knockout screening, acyl-CoA thioesterase 2 (*ACOT2*) was identified and validated as a downstream target of MI202. Knockdown of *ACOT2* partially rescued the effect of MI202 on *MYCN* expression, suggesting a potential ACOT2-dependent mechanism in regulating *MYCN* expression.

## Materials and methods

### Chemical

A core library of 9,600 representative compounds was provided from the Drug Discover Initiative (DDI, the University of Tokyo, Japan) under a blinded screening format, with restrictions on disclosing compound identities as part of the licensing agreement. MI202 (CAS No: 383135-58-8) was synthesized and purchased from MedChemExpress. ACR and its methyl derivatives ACR-55 were supplied by Kowa (Tokyo, Japan). Alectinib (CAS No: 1256580-46-7), alpelisib (CAS No: 1256580-46-7), indirubin (CAS No: 479-41-4), crizotinib (CAS No: 877399-52-5), temsirolimus (CAS No: 162635-04-3), ridaforolimus (CAS No: 572924-54-0), rapamycin (CAS No: 53123-88-9), azacitidine (CAS No: 320-67-2) and decitabine (CAS No: 2353-33-5) were purchase from Selleck (Tokyo, Japan).

## Results

### Establishment of a high-throughput screening system for *MYCN* expression inhibitor

**Figure 1:**
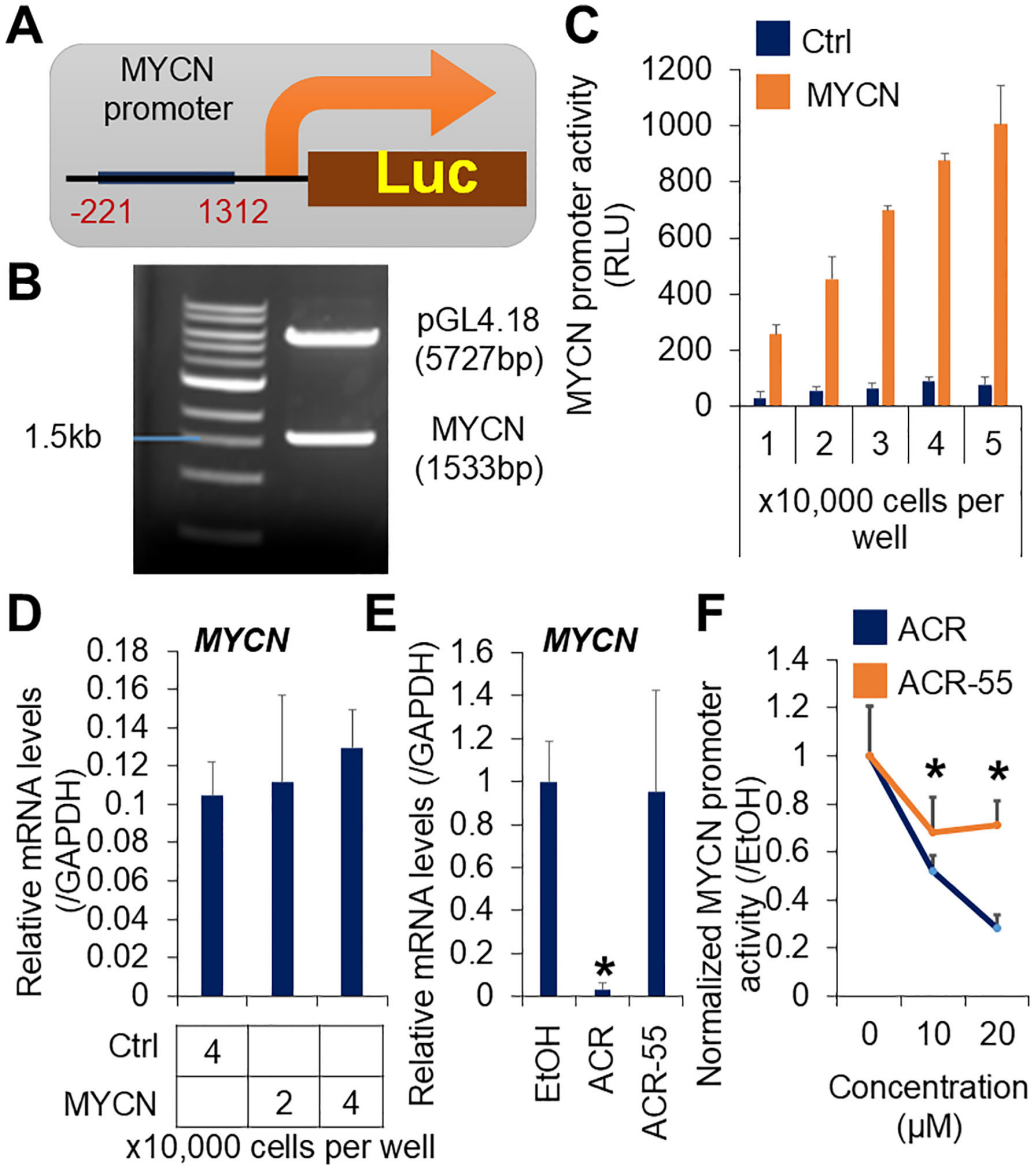
Establishment of a high-throughput screening system for *MYCN* expression inhibitor. **(A)** Schematic overview of the *MYCN* promoter integrated luciferase reporter vector. **(B)** Agarose gel electrophoresis of the *MYCN* promoter integrated luciferase reporter vector digested by SacI. **(C)** Luciferase activity and **(D)** Relative gene expression of MYCN in the JHH7 cells transfected with the *MYCN* promoter integrated and the mock (Ctrl) luciferase reporter vectors. **(E)** Relative *MYCN* expression in JHH7 MYCN-Luc stable cells treated with EtOH, ACR, or ACR-55 at 20 μM for 6 hours. **(F)** Cell viability-normalized *MYCN* promoter activity in JHH7 MYCN-Luc stable cells treated with EtOH, ACR, or ACR-55 at indicated concentrations for 24 (h) The data are presented as mean (n =3 replicates) ± SD. *P < 0.05, Student’s *t*-test.

To screen for compounds that inhibit *MYCN* expression, we developed stable reporter HCC cell lines by transfecting a luciferase reporter plasmid containing *MYCN* promoter elements (MYCN-Luc) from region -221 to +1312 (**Figure 1A**). The +1 position corresponds to the transcription initiation site, as previously described (35). The *MYCN* promoter region was subcloned into the SacI restriction site of the promoter-free pGL4.18 luciferase plasmid. SacI digestion confirmed the *MYCN* promoter band (1533 bp) and the linearized plasmid vector band (5727 bp) (**Figure 1B**). The PCR products were verified using Sanger sequencing (data not shown). The specificity and efficiency of the screening system were validated through cell number-dependent increases in *MYCN* promoter activity in JHH7 MYCN-Luc cells compared to cells transfected with the mock vector (Ctrl-Luc) (**Figure 1C**), whereas there was no obvious difference in their endogenous *MYCN* expression levels (**Figure 1D**). ACR, a known inhibitor of *MYCN* expression (36), significantly reduced *MYCN* expression (**Figure 1E**) and promoter activity (**Figure 1F**) in JHH7 MYCN-Luc cells. These effects were absent in the inactive methyl derivative of ACR, ACR-55 (**Figures 1E, F**). Overall, the JHH7 MYCN-Luc cell line represents an effective tool for high-throughput screening to identify *MYCN* expression inhibitors.

### Identification of MI202 as a novel *MYCN* expression inhibitor

**Figure 2:**
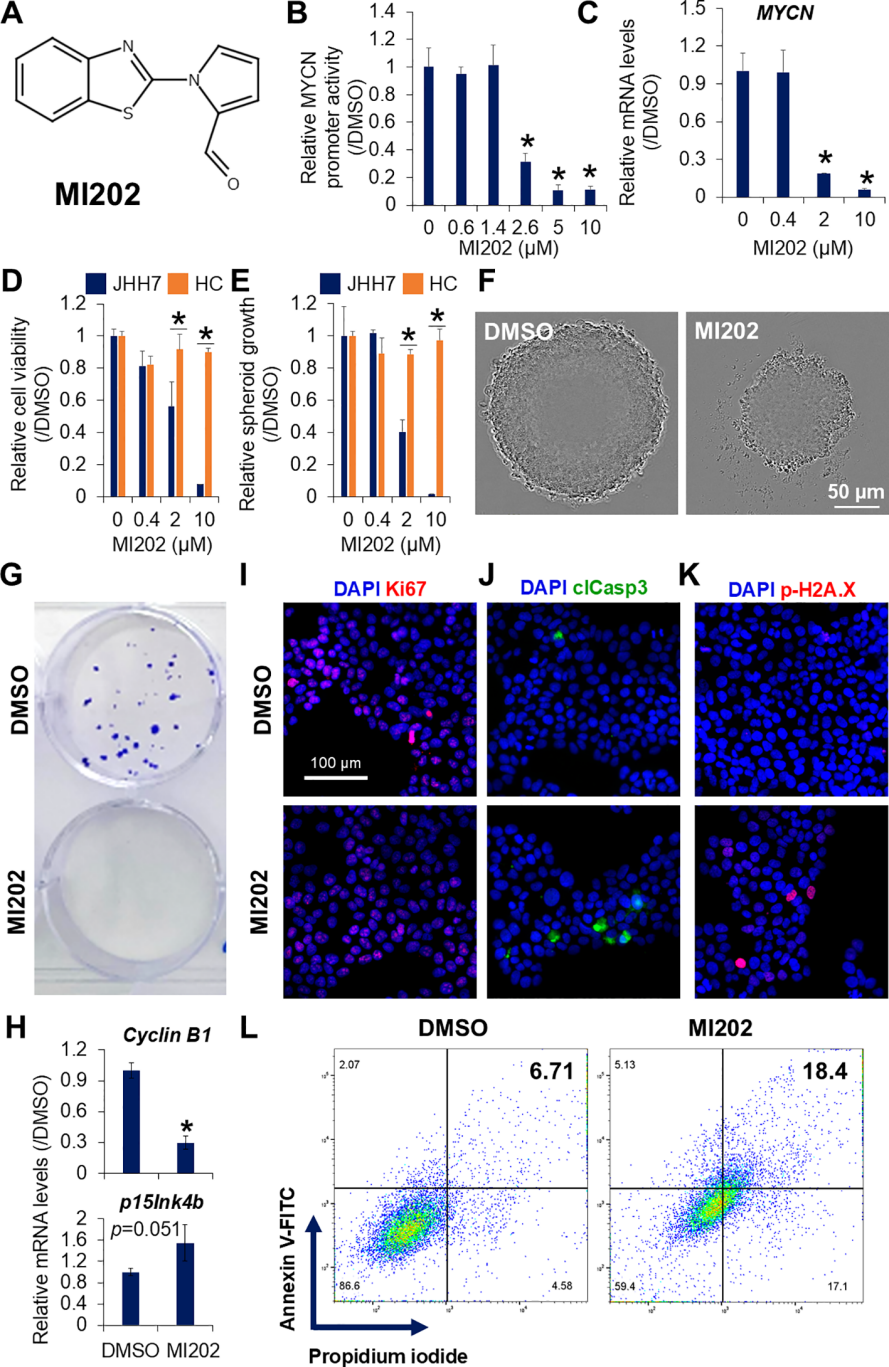
Identification of MI202 as a novel MYCN expression inhibitor. **(A)** Chemical structure of MI202. Dose-dependent inhibitory effects of MI202 on **(B)** *MYCN* promoter activity in JHH7 MYCN-Luc stable cells and **(C)** *MYCN* expression in JHH7 cells treated with MI202 at indicated concentrations for 24 and 6 hours, respectively. Dose-dependent inhibitory effects of MI202 on proliferation of JHH7 and Hc cells grown as **(D)** monolayer and **(E)** sphere cultures at indicated concentrations for 4 days. **(F)** Representative phase-contrast images of spheroid formation and **(G)** crystal violet staining of colony formation in JHH7 cells treated with DMSO or 10 μM MI202. **(H)** Relative *cyclin B1* and *p15Ink4b* expression; representative immunofluorescence staining for **(I)** cellular proliferation marker Ki67, **(J)** apoptosis marker clCasp3, and **(K)** DNA damage marker p-H2A.X; and **(L)** apoptotic cell death detected by dual staining with Annexin V-FITC and propidium iodide followed by flow cytometry in JHH7 cells treated with DMSO or 10 μM MI202 for 24 hours. *P < 0.05, Student’s *t*-test.

High-throughput screening was performed using 9,600 compounds from the DDI core library in JHH7 MYCN-Luc cells. The inhibitory effect of each compound at a single dose of 10 μM for 24 hours on *MYCN* promoter activity was examined relative to DMSO (**Supplementary Figure S1A**; **Supplementary Table S1**). Among the compounds showing a fold change greater than 2, 7 hit compounds were selected based on their dose-dependent inhibition of cell viability-normalized *MYCN* promoter activity. To evaluate whether these compounds reduced endogenous *MYCN* expression, further validation was performed and confirmed by PCR analysis. Four of these compounds inhibited *MYCN* expression in a dose-dependent manner in JHH7 cells (**Supplementary Figure S1B**). Among them, one compound, designated as MI202, exhibited favorable properties for inhibiting *MYCN* expression, positioning it as a promising candidate for suppressing HCC cell growth and proliferation. The chemical structure of MI202 is characterized by extensive conjugation with multiple double bonds and includes bioactive thiadiazole and oxadiazole rings (**Figure 2A**). MI202 inhibited both *MYCN* promoter activity (IC50 = 2.21 μM) and endogenous *MYCN* expression (IC50 = 1.07 μM) in JHH7 cells in a dose-dependent manner (**Figures 2B, C**). Consistent with its effect on MYCN mRNA expression, MI202 also suppressed MYCN protein expression in a time- and dose-dependent manner (**Supplementary Figures S2A, B**). The IC50 for MYCN promoter activity is slightly higher than that for endogenous MYCN gene expression. Several potential reasons could account for this difference, such as: 1) Differences in sensitivity between detection methods; 2) The inserted MYCN promoter region may not fully reflect the endogenous transcriptional regulatory mechanisms, especially cis-regulation from enhancers or insulators; 3) MYCN promoter activity is measured through luciferase protein expression, which is influenced not only by transcriptional regulation but also by translation processes. MI202 also reduced cell viability in JHH7 cells, both in monolayer (IC50 = 2.46 μM) and spheroid (IC50 = 1.55 μM) cultures, while no effects were observed in Hc cells (IC50 > 10 μM, respectively) (**Figures 2D, E**). Micrograph analysis confirmed the significant inhibition of sphere growth (**Figure 2F**) and colony formation (**Figure 2G**) in MI202-treated JHH7 cells. PCR revealed that MI202 suppressed the expression of the cell cycle regulator *cyclin B1* and upregulated the expression of the cyclin-dependent kinase inhibitor *p15Ink4b* in JHH7 cells (**Figure 2H**). Similarly, immunofluorescence staining revealed a decrease in the number of cells labeled with the cellular proliferation marker Ki67 (**Figure 2I**) and an increase in the number of cells labeled with the apoptosis marker clCasp3 (**Figure 2J**) in MI202-treated JHH7 cells. Notably, MI202 induced apoptosis in JHH7 cells in a time- and dose-dependent manner, as indicated by the expression levels of clCasp3 (**Supplementary Figures S2A, B**). MI202 also enhanced p-H2A.X expression (**Figure 2K**), a marker linked to apoptosis sensitivity (37). Finally, flow cytometry confirmed an increase in Annexin V-positive apoptotic cells following MI202 treatment in JHH7 cells (**Figure 2L**). These data suggest that MI202 suppresses cell proliferation and induces apoptosis in JHH7 cells. To further explore the broader effect of MI202, we evaluated its impact on additional HCC cell line Huh-1. MI202 significantly inhibited both *MYCN* expression (**Supplementary Figure S2C**) and cell proliferation (**Supplementary Figure S2D**) in Huh-1 cells.

Next, we compared the effects of MI202 with other known inhibitors targeting MYCN-driven tumors. As shown in **Supplementary Figure S3**, MI202 demonstrated superior inhibition of endogenous *MYCN* expression and cell proliferation in JHH7 cells compared to ALK inhibitors (alectinib and crizotinib), PI3K/Akt/mTOR pathway inhibitors (alpelisib and rapamycin), and DNA methyltransferase inhibitors (azacitidine and decitabine). Notably, the PI3K/Akt/mTOR inhibitors indirubin, ridaforolimus, and temsirolimus also significantly suppressed *MYCN* expression, accompanied by a marked reduction in JHH7 cell proliferation. Additionally, we investigated the combined effects of MI202 with inhibitors that significantly suppressed MYCN expression on JHH7 cell proliferation. Synergistic effects were observed when MI202 was combined with crizotinib or rapamycin on JHH7 cell proliferation. Notably, low doses of indirubin, ridaforolimus or temsirolimus alone strongly inhibited JHH7 cell proliferation. When combined with MI202, indirubin and ridaforolimus demonstrated a more pronounced inhibitory effect on JHH7 proliferation compared to either agent alone.

### Genome-wide CRISPR screening identifies *ACOT2* as a molecular target of MI202

**Figure 3:**
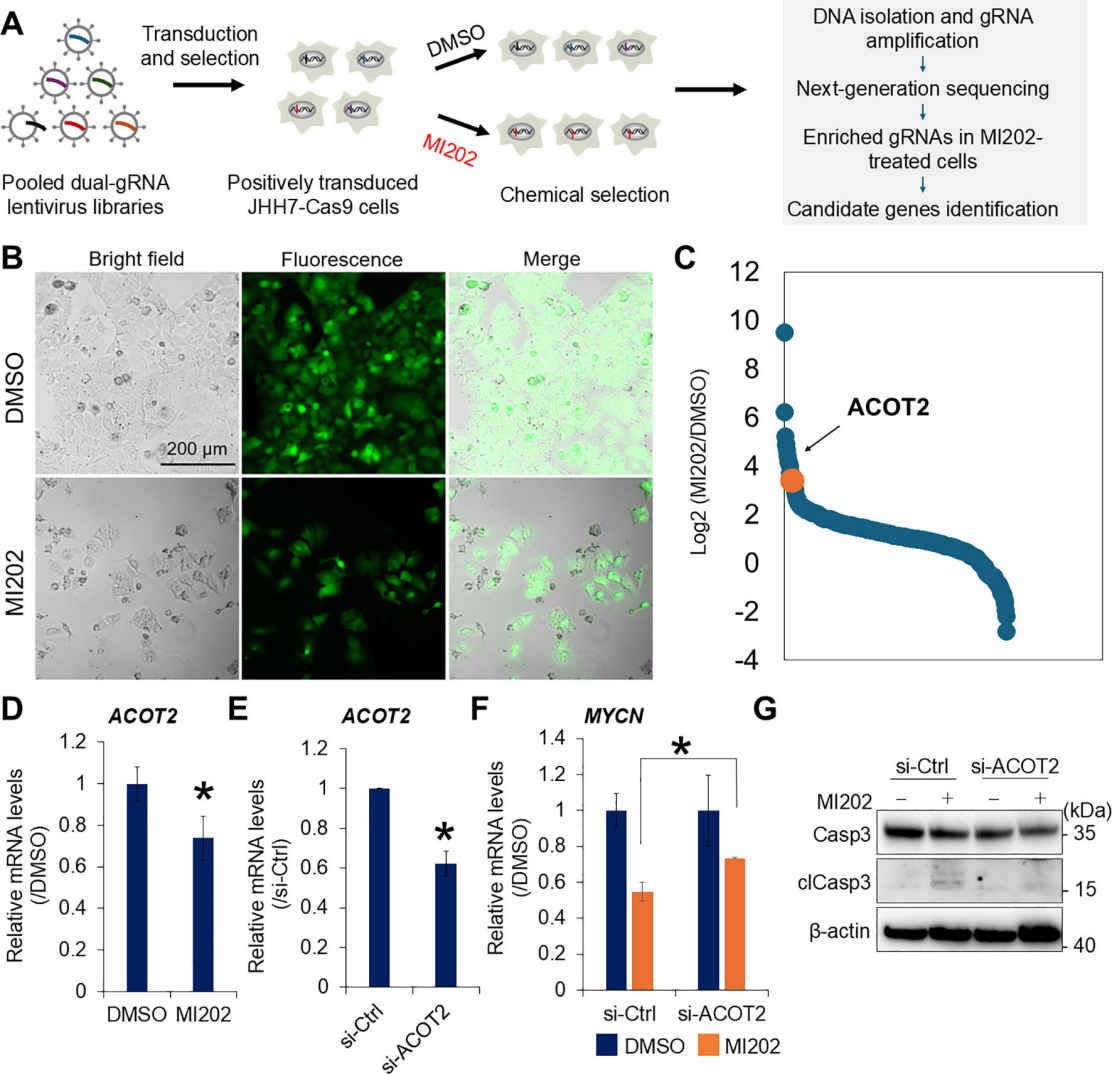
Genome-wide CRISPR screening identifies ACOT2 as a molecular target of MI202. **(A)** Schematic overview of genome-wide CRISPR knockout screening strategy. **(B)** Microscopic analysis of human Cas9-overexpressing JHH7 cells transduced with a GFP-labeled human whole genome dual-gRNA lentivirus library. Cells were selected using puromycin and treated with DMSO or 10 μM MI202 for 48 hours. **(C)** Ranking of fold change in gRNA abundance between MI202- and DMSO-treated cells. The gRNA targeting *ACOT2* is highlighted in orange. **(D)** Relative *ACOT2* expression in JHH7 cells treated with DMSO or 10 μM MI202 for 24 hours. **(E)** Relative *ACOT2* expression in JHH7 cells transfected with either si-Ctrl or si-ACOT2 for 24 hours. **(F)** Relative *MYCN* expression in si-Ctrl and si-ACOT2-transfected JHH7 cells treated with DMSO or 10 μM MI202 for 6 hours. The data are presented as mean (n =3 replicates) ± SD. *P < 0.05, Student’s *t*-test. **(G)** Western blot analysis of Casp3 and clCasp3 expression in JHH7 cells transfected with si-Ctrl or si-ACOT2 and treated with either DMSO or 10 μM MI202 for 24 hours.

To identify the genes involved in the MI202-mediated inhibition of *MYCN* expression, we performed genome-wide CRISPR knockout screening using a GFP-labeled lentiviral gRNA library in Cas9-overexpressing JHH7 cells (**Figure 3A**). Transduced cells were selected with puromycin and validated by GFP signals after 2 days of DMSO or MI202 treatment (**Figure 3B**). Genomic DNA isolated from surviving cells was analyzed using NGS to evaluate gRNA abundance. Its enrichment implies that the corresponding gene contributes to the effectiveness of MI202. gRNAs with a total count greater than 100 were selected and ranked based on their fold change between MI202- and DMSO-treated cells (**Figure 3C**; **Supplementary Table S2**). Notably, gRNAs targeting *ACOT2* were enriched in MI202-treated cells, with a fold change greater than 10 compared to those treated with DMSO, highlighting *ACOT2* as a gene of particular interest. Consistent with this, MI202 significantly downregulated *ACOT2* expression in JHH7 cells (**Figure 3D**). To investigate whether *ACOT2* plays a functional role in the effects of MI202 in JHH7 cells, we conducted a loss-of-function analysis using siRNA-mediated *ACOT2* knockdown (**Figure 3E**). Interestingly, siRNA-mediated *ACOT2* knockdown partially reversed the effect of MI202 effect on *MYCN* expression in JHH7 cells (**Figure 3F**). Furthermore, we evaluated the impact of ACOT2 on MI202-induced apoptosis in JHH7 cells. ACOT2 knockdown partially attenuated MI202-induced clCasp3 expression (**Figure 3G**). These results suggest that the inhibitory effects of MI202 on *MYCN* expression and apoptosis in JHH7 cells are, at least in part, mediated through ACOT2-mediated signaling pathways.

### *In vivo* pharmacokinetics of MI202

To support further translational and preclinical investigations, we conducted preliminary pharmacokinetic characterization of MI202 in mice. Plasma pharmacokinetics were evaluated following oral (p.o.), intravenous (i.v.), and intraperitoneal (i.p.) administration at a dose of 5 mg/kg body weight (**Supplementary Figure S3**). For p.o. administration, the maximum drug concentration (Cmax) was 18 ng/ml and the half-life (T1/2) was within 2 hours. For i.v. administration, the Cmax was 1,056 ng/ml, and the T1/2 was within 30 minutes. Plasma MI202 was undetectable following intraperitoneal administration, indicating that the intraperitoneal route may not be suitable for delivering MI202. These results demonstrate that the pharmacokinetics of MI202 are route-dependent, and further structural optimization is necessary to improve its pharmacological profile for evaluating its anti-HCC activity *in vivo*, including in xenograft models.

## Discussion

The role of liver CSCs in regulating HCC stemness, self-renewal, tumorigenicity, metastasis, recurrence, and therapeutic resistance during disease progression is well-recognized. *MYCN* expression is restricted to CSC-like HCC cells and plays a critical role in tumorigenesis (14, 16). Our previous study demonstrated that *MYCN* overexpression is associated with liver tumorigenesis and HCC progression through lipid desaturation-mediated membrane reprogramming (15) and miRNA-based signaling regulation, such as by the tumor-suppressor miR-493-5p (18). This suggests that MYCN contributes to HCC cell growth and invasion by promoting multiple pathological pathways, highlighting *MYCN* overexpression as a critical mechanism in HCC progression (9b). In this study, we hypothesized that the pharmacological downregulation of *MYCN* could represent a novel approach for inhibiting HCC cell proliferation. To this end, we conducted unbiased high-throughput screening to identify compounds capable of downregulating *MYCN* expression in HCC cells and identified MI202 as a novel *MYCN* expression inhibitor.

Our study is the first comprehensive screening of small molecule inhibitors targeting *MYCN* expression in HCC cells. We demonstrated that MI202 treatment reduced *MYCN* promoter activity, leading to decreasing *MYCN* mRNA levels in JHH7 cells. In addition, MI202 regulated the expression of genes associated with the cell cycle, promoted apoptosis, and inhibited spheroid and colony formation in JHH7 cells. MYC proteins play essential roles in stem cell renewal and tissue regeneration, which has historically led to their classification as "undruggable" due to concerns regarding potential adverse effects on normal proliferative tissues (13). Our findings revealed that MI202 selectively inhibited *MYCN* promoter activity and cell proliferation in JHH7 cells, in both monolayer and sphere cultures but not in Hc cells, suggesting that targeting *MYCN* transcription offers the potential for developing an HCC-specific therapeutic strategy.

Furthermore, through CRISPR-based whole-genome knockout screening and subsequent loss-of-function validation, we identified *ACOT2* as a target of MI202. ACOT2 is a member of the type-I acyl-CoA thioesterase family, a group of enzymes involved in lipid metabolism that catalyzes the hydrolysis of acyl-CoA into free fatty acids and coenzyme A (38). *ACOT2* is expressed in the liver, brain, and kidneys, localized within the mitochondria, and has been implicated in cancer development. Upregulation of *ACOT2* has been reported in acute myeloid leukemia (39) and breast cancer cells (40), where it is associated with poor survival outcomes. ACOT2 is also one of the genes found to be altered in the metabolome-proteome profile of lung adenocarcinoma (41). In the present study, MI202 reduced *ACOT2* expression in JHH7 cells, suggesting that high *ACOT2* expression may play a detrimental role in HCC.

**Figure 4:**
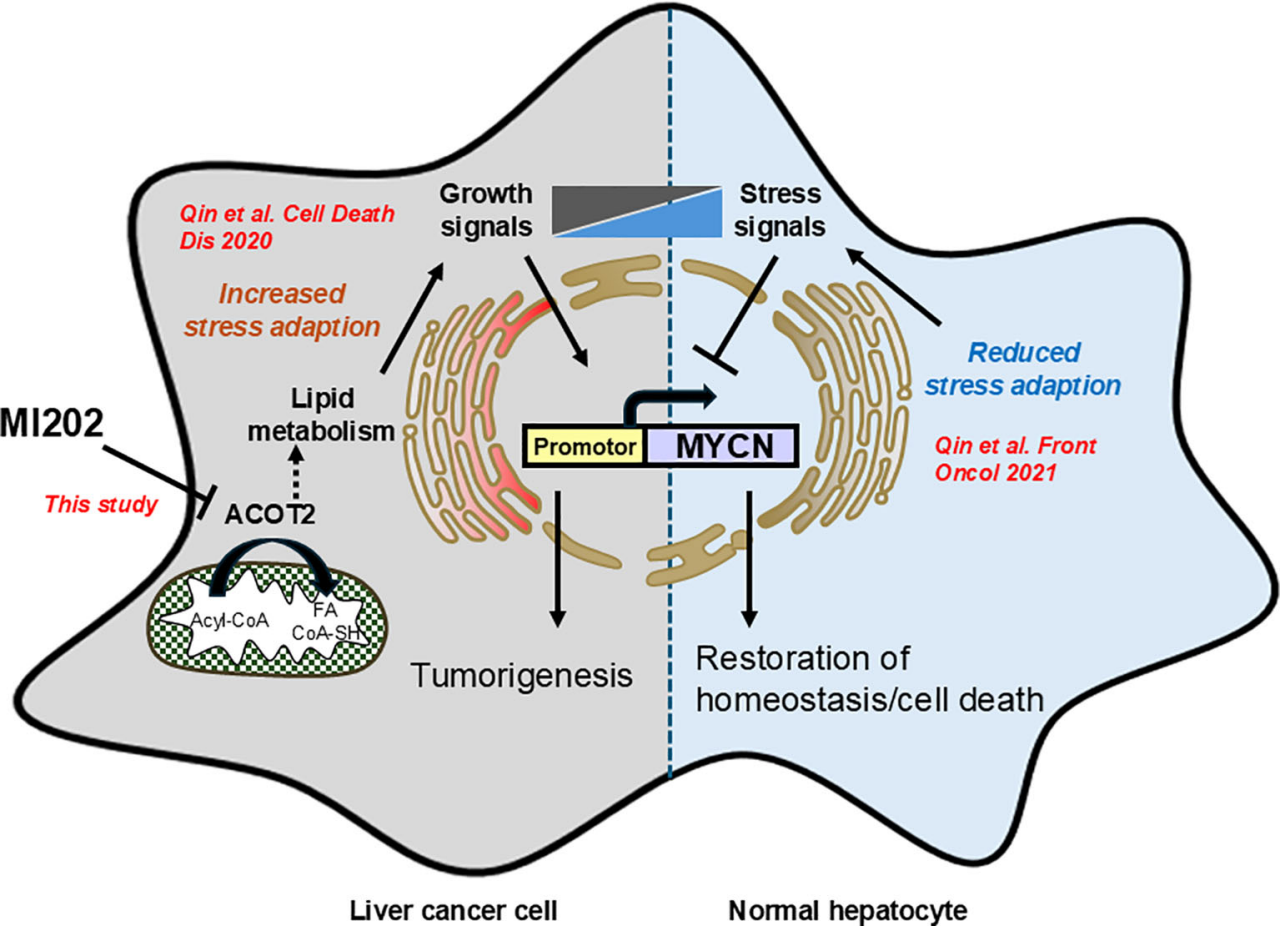
Schematic diagram illustrating the inhibitory effects of MI202 on *MYCN* expression. Our previous study demonstrated that MYCN gene expression in the inflammatory microenvironment of liver cancer is regulated by the balance between growth and stress signals, which promote and inhibit MYCN expression, respectively (9). This study identifies MI202 as an inhibitor of MYCN expression and HCC cell proliferation through ACOT2-dependent pathways. The proposed mechanism may involve lipid metabolism-associated ER stress signaling, which inhibits MYCN expression in HCC cells.

In summary, we developed an unbiased high-throughput screening platform to identify inhibitors of *MYCN* expression with potential therapeutic efficacy in HCC. Here, we identified MI202 as a novel inhibitor of *MYCN* expression, partially through the regulation of the downstream molecule *ACOT2* (**Figure 4**). MI202 inhibited cell growth, reduced colony formation, and promoted cell death in HCC cells. However, the precise mechanisms of action, as well as the optimization of the compound to improve its pharmacokinetics for *in vivo* studies require further investigation. These findings support the concept that pharmacological downregulation of *MYCN* could offer new therapeutic opportunities and potentially synergize with standard therapies and immuno-oncology approaches for HCC treatment.

## Data Availability

The original contributions presented in the study are included in the article/**Supplementary Material**. Further inquiries can be directed to the corresponding author.
