# Supplementary material for: A high-throughput screening platform to identify MYCN expression inhibitors for liver cancer therapy
Source: Front Oncol. 2025 Feb 14;15:1486671. doi: 10.3389/fonc.2025.1486671 (PMC11868045; doi:10.3389/fonc.2025.1486671)
Supplement: Supplementary file 1 [file DataSheet1.pdf]

Supporting Information

A high-throughput screening platform to identify MYCN expression inhibitors for liver cancer therapy

Yali Xu[1,2], Hricha Mishra[1], Yutaka Furutani[3], Kaori Yanaka[3], Hajime Nishimura[1], Erina Furuhata[1], Masataka Takahashi[1], Luc Gailhouste[4], Yusuke Suenaga[5], Yoshitaka Hippo[5], Wenkui Yu[2], Tomokazu Matsuura[3], Harukazu Suzuki[1], Xian-Yang Qin[1]

[1] Laboratory for Cellular Function Conversion Technology, RIKEN Center for Integrative Medical Sciences, Kanagawa, Yokohama 2300045, Japan

[2]Department of Intensive Care Unit, The Affiliated Drum Tower Hospital, Medical School of Nanjing University, Nanjing, Jiangsu 210008, China

[3]Department of Laboratory Medicine, The Jikei University School of Medicine, Tokyo 1058461, Japan

[4]Laboratory for Brain Development and Disorders, RIKEN Center for Brain Science, Saitama 3510106, Japan

[5]Laboratory of Evolutionary Oncology, Chiba Cancer Center Research Institute, Chiba

2600801, Japan

Supplementary Materials and Methods

Cell culture

Human normal hepatic cell line Hc and HCC cell lines JHH7 and Huh-1 were maintained in Dulbecco's modified Eagle's medium (ThermoFisher) supplemented with 10% fetal bovine serum (Sigma-Aldrich, Darmstadt, Germany) and 100 U/mL penicillin/streptomycin. Cells were grown at 37 °C in a humidified incubator under 5% CO_2.

Whole-genome dual-gRNA CRISPR knockout screening

A human Cas9-overexpressing JHH7 cell clone was generated using a Mammalian Cas9 Expression PiggyBac Vector (VectorBuilder, Yokohama, Japan). The cells were transduced with a GFP-labeled human whole-genome dual-gRNA lentiviral library (VectorBuilder) at a multiplicity of infection of 10. Antibiotic selection was initiated 5 days post-infection using 10 µg/ml puromycin. Cells were then cultured in DMSO or 10 µM MI202 for 48 h. Genomic DNA was extracted from surviving cells using the NucleoSpin Tissue kit (Takara). The PCR amplicons were analyzed via next-generation sequencing (NGS) using NovaSeq 2×150 bp sequencing (Illumina, San Diego, CA, USA). Changes in gRNA abundance were examined between DMSO and MI202 treatment groups.

RNA isolation and real-time PCR

Total RNA was extracted using the FastGene RNA Basic Kit (NIPPON Genetics, Tokyo, Japan) and RNA quantity and purity were determined using a Nanodrop spectrophotometer (Thermo Fisher Scientific, Waltham, MA, USA). cDNA synthesis was performed using the PrimeScript RT Master Mix Kit (Takara, Otsu, Japan). PCR reactions were conducted using the SYBR Premix ExTaq II (Takara) on a StepOne Real-Time PCR System (Applied Biosystems, Carlsbad, CA, USA). Primer sequences used in this study are as follows: β-actin (*ACTB*; forward: 5'-GCACAGAGCCTCGCCTT-3' and reverse: 5'-GTTGTCGACGACGAGCG-3'); *MYCN* (forward: 5'-TCCATGACAGCGCTAAACGTT-3' and reverse: 5'-GGAACACACAAGGTGACTTCAACA-3'); glyceraldehyde-3-phosphate dehydrogenase (*GADPH*; forward: 5'-CCATGGAGAAGGCTGGGG-3' and reverse: 5'-CAAAGTTGTCATGGATGACC-3'); *ACOT2* (sense: 5'-GCCCGAGAGGATGTCTAACA-3' and reverse: 5'-TCAGGCTCCATTGGTACAGC-3'); cyclin B1 (forward: 5'-CGGGAAGTCACTGGAAACAT-3' and reverse: 5'-AAACATGGCAGTGACACCAA-3'); cyclin dependent kinase inhibitor 2B (*p15Ink4b*; forward: 5' CCCAACTCCACCAGATAGCA-3' and reverse: 5'-GGGATTTCCGCATCCT AGCA-3'). The housekeeping genes *GAPDH* or *ACTB* were

used as an internal standard.

siRNA transfection

Silencer® Select pre-designed siRNA targeting human *ACOT2* (4427037, si-ACOT2) and control siRNA (4390844, si-Ctrl) were obtained from Thermo Fisher Scientific. Cells were transfected with 5 nM siRNA using Lipofectamine 3000 transfection reagent (Thermo Fisher Scientific) at a concentration of 0.75 μL/well in 24-well plates for further analysis.

Cell viability assay

Cell viability was examined using the Cell Counting Kit-8 (Dojindo Molecular Technologies, Tokyo, Japan) according to the manufacturer's instructions. Absorbance was measured at 450 nm using the SpectraMax iD5 microplate reader (Molecular Devices, San Jose, CA, USA).

Spheroid proliferation assays

3D spheroid cultures were established in non-adherent 96-well round-bottomed Sumilon PrimeSurface plates (MS-9096U, Sumitomo Bakelite, Tokyo, Japan) at 5,000 cells per well. Spheroids were cultured for 4 days, and images were captured using an optical microscope (ZEN, NIKON, Tokyo, Japan). Spheroid proliferation was assessed using the CellTiter-Glo Cell Viability Assay (Promega Corporation, Madison, WI, USA) on the

SpectraMax iD5 microplate reader (Molecular Devices).

Clonogenic assay

Cells were seeded in six-well plates at a density of 400 cells per well. On the second day, cells were treated with DMSO or 10 μM MI202. Fresh medium containing the compounds was replaced every 4 days for a total of 12 days. Cells were washed twice with PBS and stained with 0.5% crystal violet for 10 minutes.

Real-time cell confluency analysis

Cells were seeded in 96-well plates at a density of 10,000 cells per well. On the second day, cells were treated with DMSO, MI202, ALK inhibitors (alectinib and crizotinib), PI3K/Akt/mTOR inhibitors (indirubin, ridaforolimus, temsirolimus, alpelisib and rapamycin), and epigenetic modulators (azacitidine and decitabine) at concentrations of 2 μM and 10 μM. Cell images were captured at 1, 2, and 3 days post-treatment, and cell confluency was calculated using the Incucyte Zoom system.

Immunofluorescence staining

JHH7 cells grown on Greiner 96-well microtiter plates (Greiner Bio One) were fixed with 4% paraformaldehyde for 10 min and permeabilized with 0.5% Triton X-100 in PBS for 10 min. Following blocking in 5% normal donkey serum in PBS plus 0.5% Triton-X for 1 h at room temperature, cells were incubated with the following primary antibodies:

cleaved caspase-3 (clCasp3, 1:200 dilution, 9661S, Cell Signaling Technology), ki67 (1:200 dilution, 350502, BioLegend), phospho-histone H2A.X (p-H2A.X, 9718, dilution 1:200 dilution, Cell Signaling Technology) at 4 °C overnight. After washing with PBS, cells were incubated with fluorescence-conjugated secondary antibodies at room temperature for 1 h. Nuclei were stained using DAPI (1:2000 dilution, Dojindo Molecular Technologies, Rockville, MD, USA). Images were acquired using a Zeiss LSM 700 laser scanning confocal microscope (Carl Zeiss Inc., Oberkochen, Germany).

Western blotting

Cells were lysed using a protein extraction reagent (ThermoFisher). Protein concentration was determined with the BCA kit (TaKaRa Bio, Kusatsu, Japan). Equal amounts of protein were loaded onto 5-20% polyacrylamide gradient gels (ATTO Inc., Tokyo, Japan) and transferred to PVDF membranes (Merck Millipore). The transferred membranes were blocked with bullet blocking one (Nacalai tesque Inc., Kyoto, Japan) at room temperature for 5 min, followed by overnight incubation at 4°C with primary antibodies: anti-MYCN (1:200, Santa Cruz Biotechnology, sc-53993), anti-Caspase 3 (Casp3; 1:1000, Biovision, 3004-100), anti-cleaved Caspase 3 (clCasp3; 1:500, Cell Signaling Technology, 9661S), and anti-β-actin (1:1000, Cell Signaling Technology, 3700). The membranes were then incubated with HRP-conjugated secondary antibodies (1:5000 dilution), protein signals

were visualized using the ECL Prime Western Blotting Detection Reagent (Cytiva).

Flow cytometry

JHH7 cells treated with DMSO or 10 μM MI202 for 24 h were harvested, stained with an Annexin V-FITC/PI apoptosis detection kit (BD Biosciences, San Jose, CA, USA) at room temperature for 15 min. Stained cells were analyzed by flow cytometry using a a FACSAriaII SORP (BD Biosciences, Franklin Lakes, NJ, USA). The data were further analyzed in FlowJo software (TreeStar, Ashland, OR, USA).

Pharmacokinetics analysis

In vivo pharmacokinetics of MI202 was evaluated in 6-week-old male C57BL/6J mice. All animal experiments were approved by the Institutional Animal Care and Use Committee of the Jikei University School of Medicine and RIKEN. The animals were maintained in a temperature-controlled and light-controlled environment with free access to standard diet and water. Mice were administered with MI202 via oral (p.o.), intraperitoneal (i.p.), and intravenous (i.v.) at a dose of 5 mg/kg in a final volume of 100 μL. Solvents used were as follows: corn oil (p.o.); 10% 2-hydroxypropyl-β-cyclodextrin and 10% PEG300 (i.v.); 5% DMSO in saline (i.p.). Calibration standard solutions were prepared by combining one volume of MI202 dilution series (0, 1, 2, 4, 10, 20 μg/ml) with nine volumes of calibration plasma. Plasma samples were collected at

0, 0.5, 1, 3, 6 h post-MI202 treatment and analyzed using the LC-MS/MS with established standard curve.

Statistical analysis

Quantitative data were expressed as mean ± standard deviation (SD) of at least three biological replicates. Statistical comparison between groups was assessed using the Student's *t*-test, with $p < 0.05$ as the level of significance.

Supplementary Discussion

The present study primarily focuses on the effect of MI202 on *MYCN* expression and its selective impact on the proliferation of HCC cells, without affecting normal hepatocytes. Although MYCN gene expression is low under steady-state conditions in hepatocytes (Qin and Gailhouste 2020), MYCN is highly expressed in stem cells and regenerative cells and is situated downstream of multiple signaling pathways in different developing organs and tissues (Nishio, et al. 2024; Ruiz-Pérez, et al. 2017). Additionally, it is important to note that ACOT2 plays a physiological role in processes such as adipocyte differentiation and cellular energy homeostasis, both of which are crucial for thermogenesis and may influence aging-related processes (Momose, et al. 2011; Widjaja, et al. 2024). This underscores the importance of considering the potential side effects of MYCN-targeted therapy in cancers, as its regulators, including ACOT2 and lipid metabolism, are implicated in tissue regeneration and healthy aging. Another limitation of our study is the lack of investigation into the effects of MI202 on other cell types, such as those within the nervous system. Furthermore, the specific regulatory mechanisms of ACOT2 on *MYCN* expression should be elucidated. The functional interplay between ACOT2 and *MYCN* expression could provide a potential therapeutic strategy for targeting ACOT2 in tumor cells.

Given the role of ACOT2 in lipid metabolism, its dysregulation may contribute to the lipid metabolic reprogramming commonly observed in HCC. Notably, aberrant lipid metabolism, including altered mitochondrial lipid composition, has been reported in patient-derived HCC tissues compared to healthy liver (Vasseur and Guillaumond 2022). Arachidonic acid (AA), an unsaturated fatty acid with pathogen-like properties and pro-inflammatory precursor, plays a pivotal role in promoting inflammation during hepatic tumorigenesis (Koundouros and Poulogiannis 2020; Kuwata and Hara 2019; Qin, et al. 2016; Qin, et al. 2018a). In this context, ACOT2 may facilitate the localized release of AA in mitochondria by hydrolyzing arachidonyl-CoA, thereby contributing to the regulation of HCC cell proliferation and tumor progression. Elevated expression levels of *ACOT8,* another member of ACOT family, have been observed in HCC clinical specimens, and *ACOT8* knockdown inhibits the growth of HCC cells by altering free fatty acid metabolism (Hung, et al. 2014). These findings highlight the crucial role of lipid metabolism in HCC development and suggest the potential prognostic impact of high *ACOT2* expression in HCC. MYCN amplification is a hallmark of aggressive neuroblastoma and is also implicated in certain medulloblastoma subtypes, targeting lipid metabolism pathways via ACOT2 inhibition could holds potential for reducing MYCN-driven tumor proliferation in these cancers. However, ACOT2 inhibition may also affect

normal brain lipid metabolism, particularly during brain development in pediatric patients (Fernández-García, et al. 2023). Our previous research demonstrated that the suppression of lipid desaturation reduces cell proliferation and downregulates *MYCN* expression in HCC cells (Qin, et al. 2020). It is consistent with the concept that targeting desaturase activity presents a potential strategy to selectively disrupt the metabolic activity of cancer cells (Peck and Schulze 2016b). ACOT2 may modulate the pool of free fatty acids available for desaturation, indirectly contributing to the regulation of *MYCN* expression. Mitochondrial ribosomal protein L19 (MRPL19), our top gene candidate in the gRNA enrichment analysis, is involved in the synthesis of mitochondrial-encoded proteins. A study by Borankova *et al.* demonstrates the role of the mitochondrial ribosome in regulating MYCN expression and inducing cancer cell death (Borankova, et al. 2023). Therefore, the mitochondrial translation machinery may also represent a downstream target of MI202 in the suppression of *MYCN* expression.

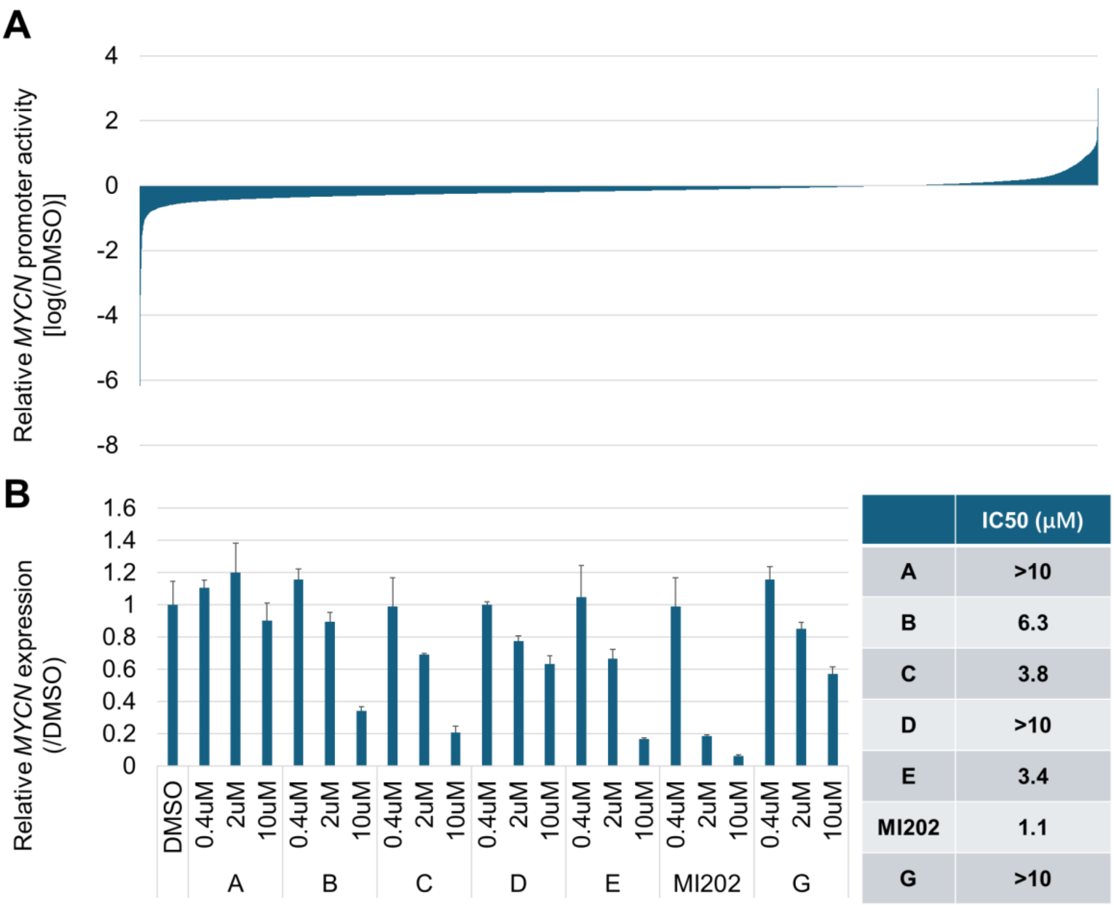

	IC50 (μM)
A	>10
B	6.3
C	3.8
D	>10
E	3.4
MI202	1.1
G	>10

Fig. S1. High-throughput screening of 9,600 compounds from the DDI core library.

(A) Inhibitory effect of each compound at a single dose of 10 μM for 24 hours on *MYCN* promoter activity. (B) Dose-dependent inhibitory effect of seven hit compounds at indicated concentrations for 6 hours on *MYCN* expression in JHH7 cells.

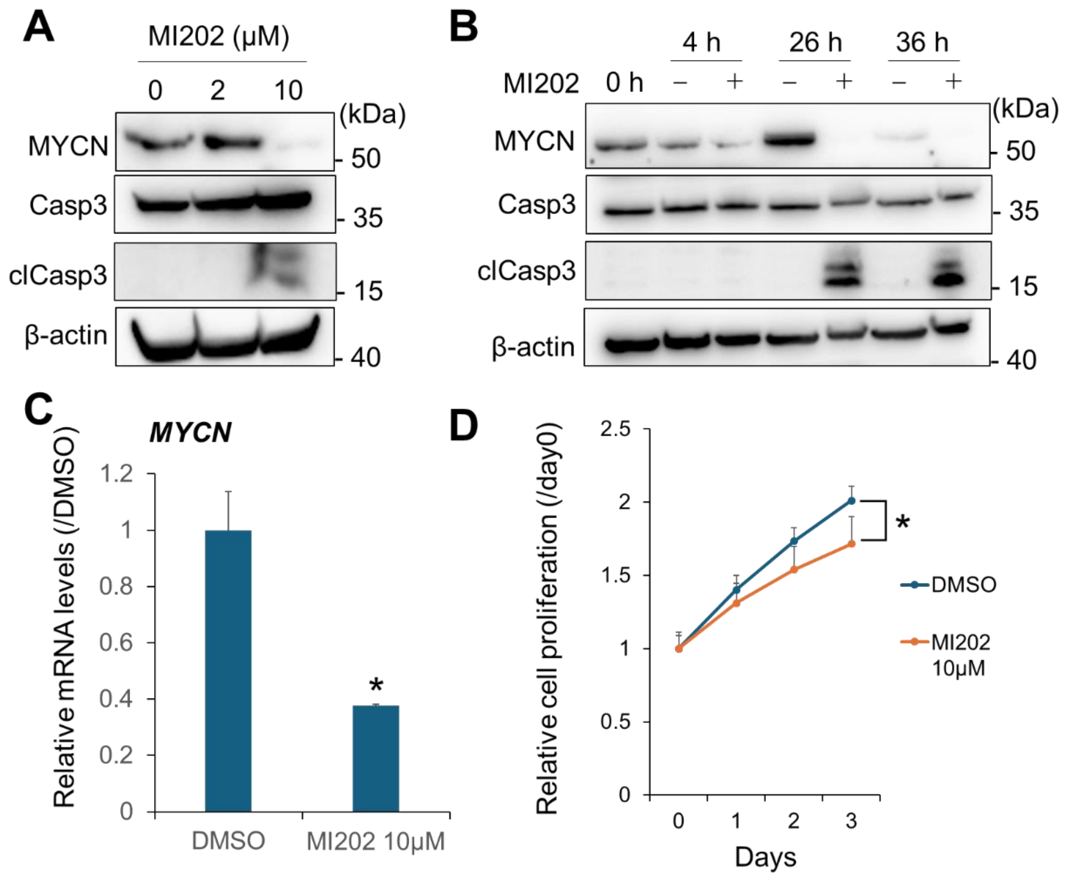

Fig. S2. Time- and dose-dependent effects of MI202 on MYCN and clCasp3 protein expression in JHH7 cells and its impact on *MYCN* expression and cell proliferation in Huh-1 cells. (A) Dose-dependent effect of MI202 on MYCN, Casp3, and clCasp3 protein expression. JHH7 cells was treated with the MI202 at 2 µM or 10 µM for 24 hours. (B) Time-dependent effect of MI202 on MYCN, Casp3, and clCasp3 protein expression. JHH7 cells was treated for the indicated times in the absence or presence of 10 µM MI202. (C) Relative *MYCN* expression in Huh-1 cells treated with DMSO or 10 µM MI202 for 24 hours. *$P < 0.05$, Student's t-test. (D) Relative cell proliferation rate of Huh-1 cells treated with DMSO or 10 µM MI202 for 3 days. *$P < 0.05$, two-way ANOVA.

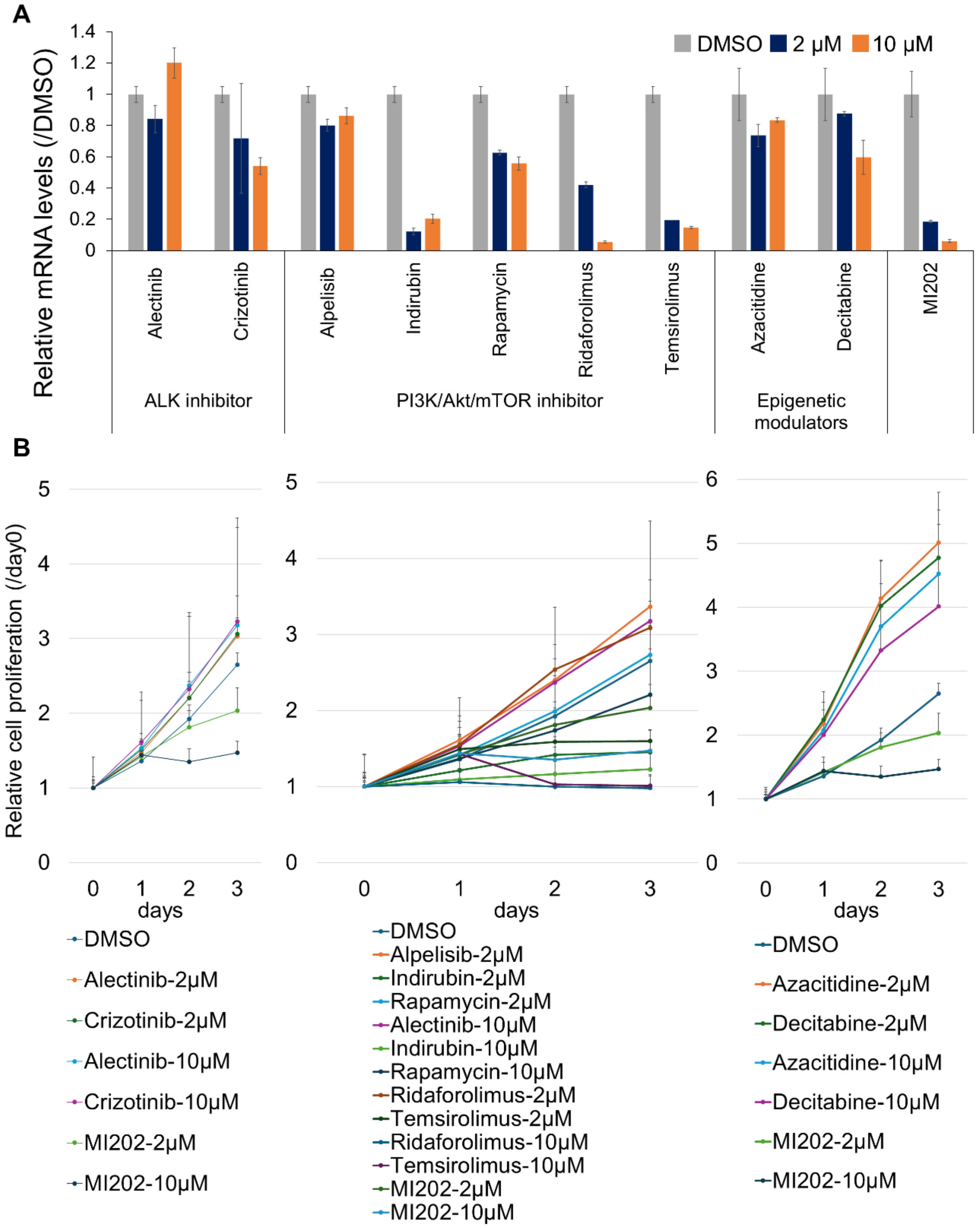
A
Relative mRNA levels (/DMSO)
DMSO
2 µM
10 µM
1.4
1.2
1
0.8
0.6
0.4
0.2
0
Alectinib
Crizotinib
Alpelisib
Indirubin
Rapamycin
Ridaforolimus
Temsirolimus
Azacitidine
Decitabine
MI202
ALK inhibitor
PI3K/Akt/mTOR inhibitor
Epigenetic modulators
B
Relative cell proliferation (/day0)
days
DMSO
Alectinib-2µM
Crizotinib-2µM
Alectinib-10µM
Crizotinib-10µM
MI202-2µM
MI202-10µM
DMSO
Alpelisib-2µM
Indirubin-2µM
Rapamycin-2µM
Alectinib-10µM
Indirubin-10µM
Rapamycin-10µM
Ridaforolimus-2µM
Temsirolimus-2µM
Ridaforolimus-10µM
Temsirolimus-10µM
MI202-2µM
MI202-10µM
DMSO
Azacitidine-2µM
Decitabine-2µM
Azacitidine-10µM
Decitabine-10µM
MI202-2µM
MI202-10µM

C

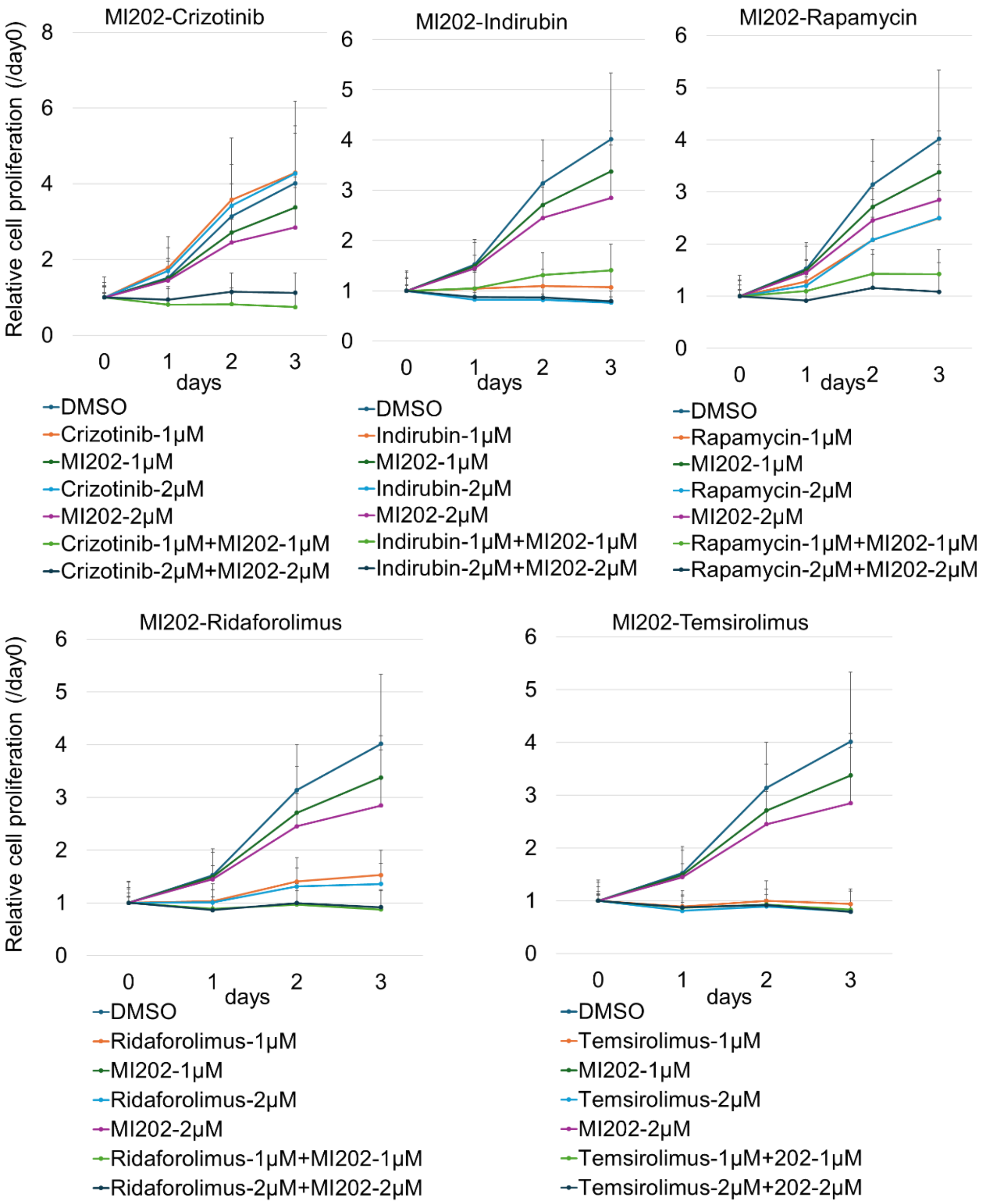

Fig. S3. Effects of ALK inhibitors, PI3K/Akt/mTOR inhibitors, and epigenetic modulators on MYCN gene expression and cell proliferation in JHH7 cells. (A) Relative MYCN gene expression in JHH7 cells following treatment with the compounds at indicated concentrations for 6 hours. (B-C) Relative cell proliferation rate of JHH7 cells after treatment with the compounds at indicated concentrations for 3 days.

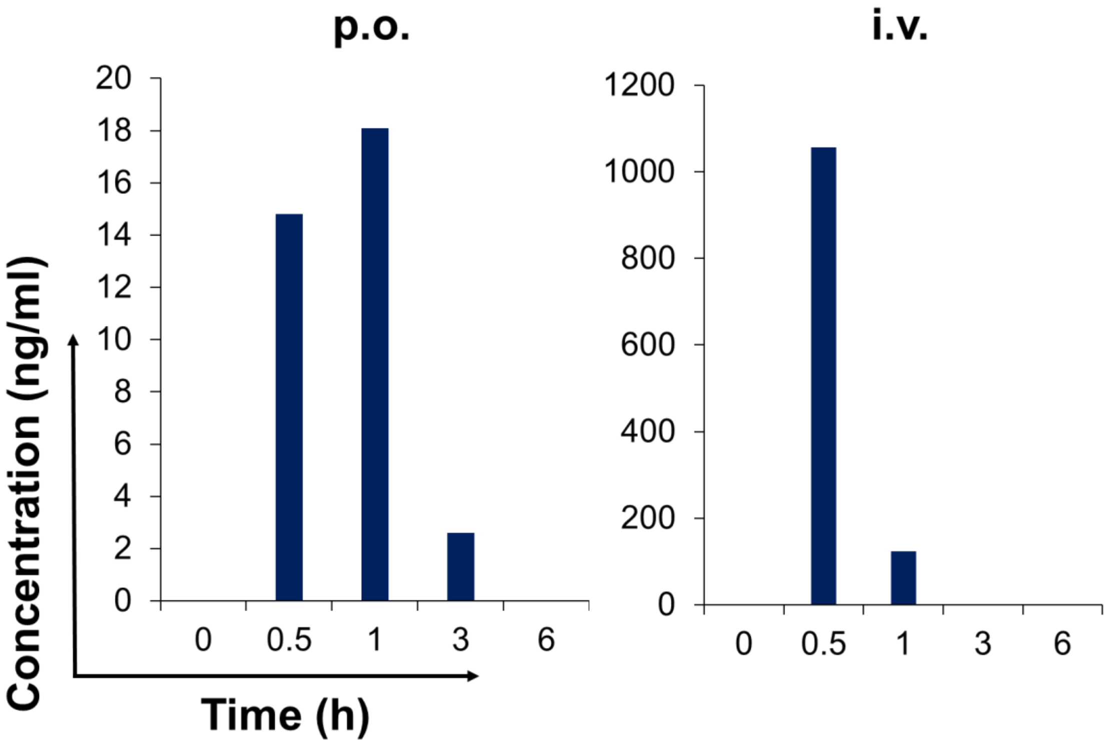

Fig. S4. **In vivo pharmacokinetics of MI202**. Plasma concentration of MI202 following oral (p.o.) and intravenous (i.v.) administration in C57BL/6 mice.

Supplementary References

Momose, Atsushi, et al.

2011 Regulated expression of acyl-CoA thioesterases in the differentiation of cultured rat brown adipocytes. Biochemical and Biophysical Research Communications 404(1):74-78.

Nishio, Yosuke, et al.

2024 MYCN in human development and diseases. Frontiers In Oncology 14:1417607.

Qin, X. Y., and L. Gailhouste

2020 Non-Genomic Control of Dynamic MYCN Gene Expression in Liver Cancer. Front Oncol 10:618515.

Ruiz-Pérez, María Victoria, Aine Brigette Henley, and Marie Arsenian-Henriksson

2017 The MYCN Protein in Health and Disease. Genes 8(4).

Widjaja, Anissa A., et al.

2024 Inhibition of IL-11 signalling extends mammalian healthspan and lifespan. Nature 632(8023):157-165.
